# Comparison of pre-labelled primers and nucleotides as DNA labelling method for lateral flow detection of *Legionella pneumophila* amplicons

**DOI:** 10.1038/s41598-024-55703-4

**Published:** 2024-02-29

**Authors:** Christian Warmt, Jette Nagaba, Jörg Henkel

**Affiliations:** Fraunhofer Institute for Cell Therapy and Immunology - Bioanalytics and Bioprocesses (IZI-BB), 14476 Potsdam, Germany

**Keywords:** Lab-on-a-chip, Isolation, separation and purification, PCR-based techniques

## Abstract

Labelling of nucleic acid amplicons during polymerase chain reaction (PCR) or isothermal techniques is possible by using both labelled primers and labelled nucleotides. While the former is the widely used method, the latter can offer significant advantages in terms of signal enhancement and improving the detection limit of an assay. Advantages and disadvantages of both methods depend on different factors, including amplification method, detection method and amplicon length. In this study, both methods for labelling PCR products for lateral flow assay (LFA) analysis (LFA-PCR) were analysed and compared. It was shown that labelling by means of nucleotides results in an increase in label incorporation rates. Nonetheless, this advantage is negated by the need for post-processing and competitive interactions. In the end, it was possible to achieve a detection limit of 3 cell equivalents for the detection of the *Legionella*-DNA used here via primer labelling. Labelling via nucleotides required genomic DNA of at least 3000 cell equivalents as starting material as well as an increased personnel and experimental effort.

## Introduction

In the days of the 2020–2023 COVID-19 pandemic, two important terms for fighting the pathogen itself, but also for controlling the pandemic, were used not only by the scientific community, but also by the mainstream press. These terms were: PCR test and rapid antigen test^1–3^. Even though these two terms have gained in importance and popularity during the pandemic, they are of course not new and corresponding methods have existed for decades for pathogen detection such as *Legionella pneumophila*^4–7^. In detail, one of these two strategies is a specialised laboratory-based analysis using nucleic acid detection and the other is an antigen detection, mostly of pathogens, based on a lateral flow test procedure^8–10^. Both methods have had their respective advantages and disadvantages for decades and thus their justification in different fields of application. For example, PCR as NAAT (Nucleic Acid Amplification Technology) is mainly used in laboratories due to its complexity and requirements for trained staff, but also due to its extremely high sensitivity and reliability. In contrast, simple lateral flow assays (LFA) can be carried out by non-professionals in the privacy of one’s own home, are less expensive and provide a quick result, as the use of pregnancy tests and rapid corona tests still impressively demonstrates^11–14^.

In recent years, and especially since isothermal amplification methods have eliminated the need for well-equipped laboratories when it comes to nucleic acid amplification, these two methods are being increasingly combined^15–21^. The result is a nucleic acid-based lateral flow detection which unites the essential advantages of both methods. On the one hand, amplification, whether PCR-based or isothermal, allows a much more sensitive assay. On the other hand, the results can be obtained by less trained personnel and, at least in theory, from anywhere in the world without the need for expensive equipment^18^.

Also, both methods individually or in combination offer a significant advantage compared to conventional microbiological tests, which are often preceded by days of cultivation^22,23^.

The use of amplification methods for the detection of *Legionella*, for example, is becoming increasingly popular, as they are extremely sensitive and can provide results within a few hours^24–26^. In addition, they can be used automatically and on site at the Point-of-Care (PoC)^27,28^.

The use of LFA test strips as downstream detection method is of interest to a number of users, particularly in terms of required equipment and involved costs. Moreover, for a large number of applications, quantitative methods such as qPCR are not necessary and the costs for technology and trained personnel can easily be saved. For example, a purely qualitative statement of the amplification as to whether contamination with *Legionella* is present can already be achieved with a simple PCR and is quite sufficient for an initial assessment of the situation. The detection of the PCR products is then possible, for example, by using these lateral flow assays. These are characterised by their quick and simple feasibility as well as straightforward interpretation of the results, for which no specialised professional training is required^29^.

Horng et al., already demonstrated a *Legionella* detection using this method in 2006 and were able to successfully detect *Legionella spp.* and *L. pneumophila* via sequences of the 16S rDNA and the *dnaJ* gene using multiplex-PCR and a lateral flow system.

**Figure 1:**
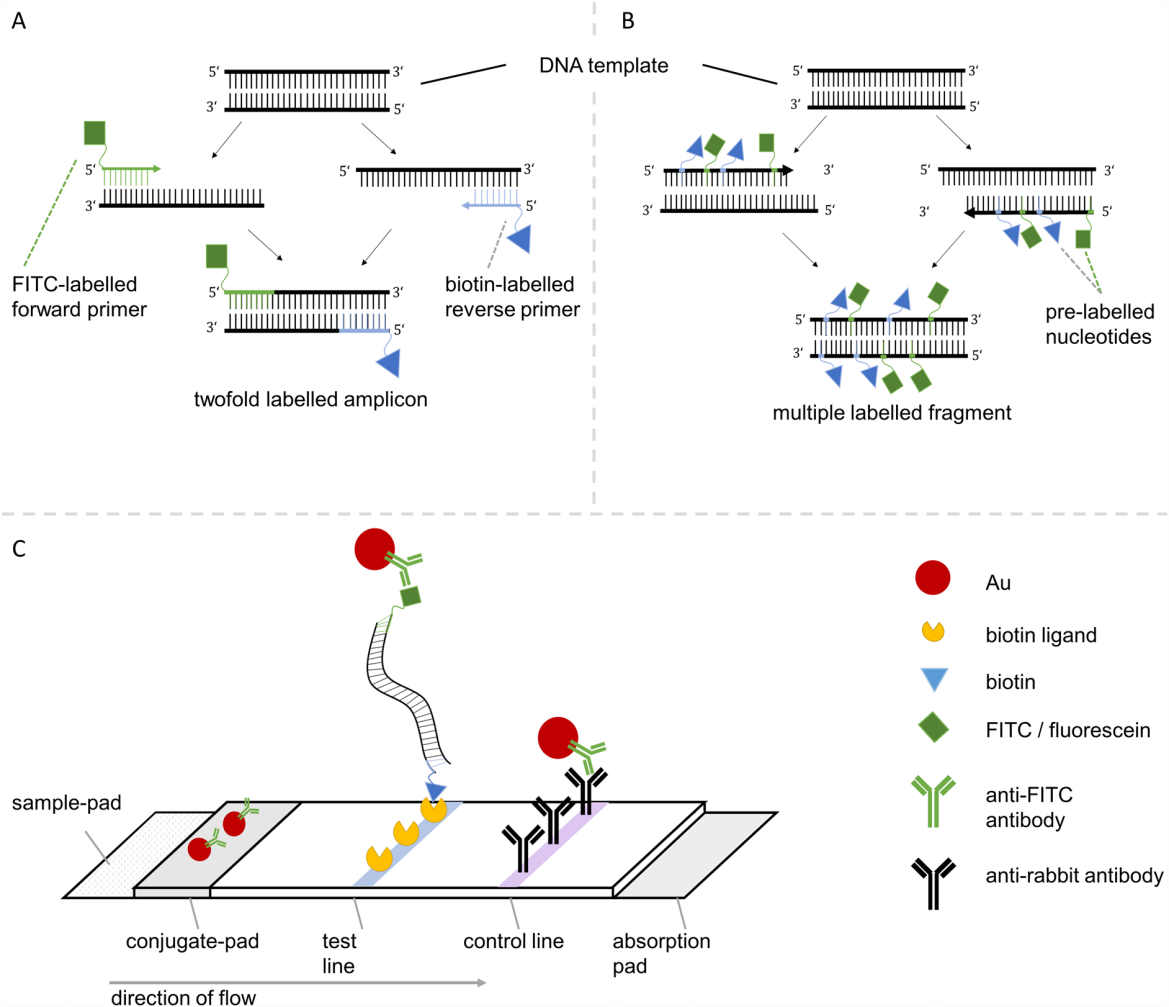
Illustration of the amplification product labelling and subsequent detection on an antibody-based lateral flow assay. (**A**) Preparation of dual-labelled PCR product using FITC- and biotin-conjugated primers. (**B**) Labelling of the PCR product by incorporation of pre-labelled nucleotides (e.g. fluorescein and biotin conjugated dUTP). (**C**) Detection of the dual labelled primer-derived amplicon via lateral flow assay as a general example for the LFA detection of dual-labelled amplification products. Detection is achieved by the binding of anti-FITC antibodies to the fragment labelled with FITC and the subsequent binding of the biotin via biotin ligands on the LFA test line. Gold nanoparticles (Au) attached to the anti-FITC antibody are used to make the nucleic acids visible.

For the detection of nucleic acids using a lateral flow method, the corresponding amplicons usually have to be labelled with two different biomolecules. There are also variations of this method where it is possible to label with just one molecule^30^ or with biotinylated capture probes^31^, but the most commonly used method is to use two different labels. One of the two is used for immobilisation on the surface, while the other is detected by a labelled antibody and represents the actual test result optically. In most cases, these two molecules are biotin or digoxigenin for immobilisation and FITC for detection^32,33^ (Fig. 1).

While pre-labelled primers are largely used for these, as well as for amplicon labelling in general^20,33–35^, a second way, i.e. labelling via nucleotides, is also conceivable and is used in various applications such as for downstream microarray analyses^36^, intercalation-induced supercoiling of DNA (ISD)^37^ or LFA-detection after isothermal amplification^38^.

In earlier studies, we were able to show that the use of labelled nucleotides can indeed result in an improvement in the labelling efficiency and thus in the sensitivity of the assays^39,40^. The fact that the amplicons can be labelled several times by using nucleotides in contrast to the usage of labelled primers (only one single molecule can be integrated) plays a major role in this case (Fig. 1A). This advantage is particularly useful in methods where the detection is realised in some way via fluorescent molecules and in this way the fluorescence signal increases proportionally to the integration rate^41–43^. However, not all applications work better this way. In a LAMP amplification, it was therefore useful to label the amplicons with FITC-primer instead of FITC-dUTP in order to be able to detect them subsequently on the LFA test strip^44^.

We analysed more than 100 PCR-LFA-based publications from 1996 to 2023 and found only one that dealt with the labelling of amplicons by biotinylated nucleotides in combination with this detection method^30^. Kortli et al., optimised their isothermal LFA assay by multiple labelling with biotin-dUTP and were able to improve the sensitivity compared to primer labelling. However, the assay used differs significantly from the currently commonly used assays and is therefore difficult to transfer to this question. To date, there is a lack of detailed analysis in the literature as to whether and to what extent the use of labelled nucleotides for LFA analysis after nucleic acid amplification, such as the PCR-LFA shown here, also provides an advantage. This gap is to be closed with this work on the basis of the detection of *Legionella* genomic DNA by means of PCR lateral flow assay (LFA-PCR). For this purpose, we investigate in detail the use of primers and nucleotides as well as a mixture of the two, and compare them both with regard to the theoretical limits of detection of the LFA strips used and the actual limits of detection (LOD) of the overall assay. In addition, aspects relating to the handling, the extent of necessary preparatory and follow-up work are discussed in order to subsequently clarify whether the use of nucleotides and thus the turning away from the previously established labelling method is worthwhile.

## Results

### Use of labelled primer for the LFA: the standard procedure

**Figure 2:**
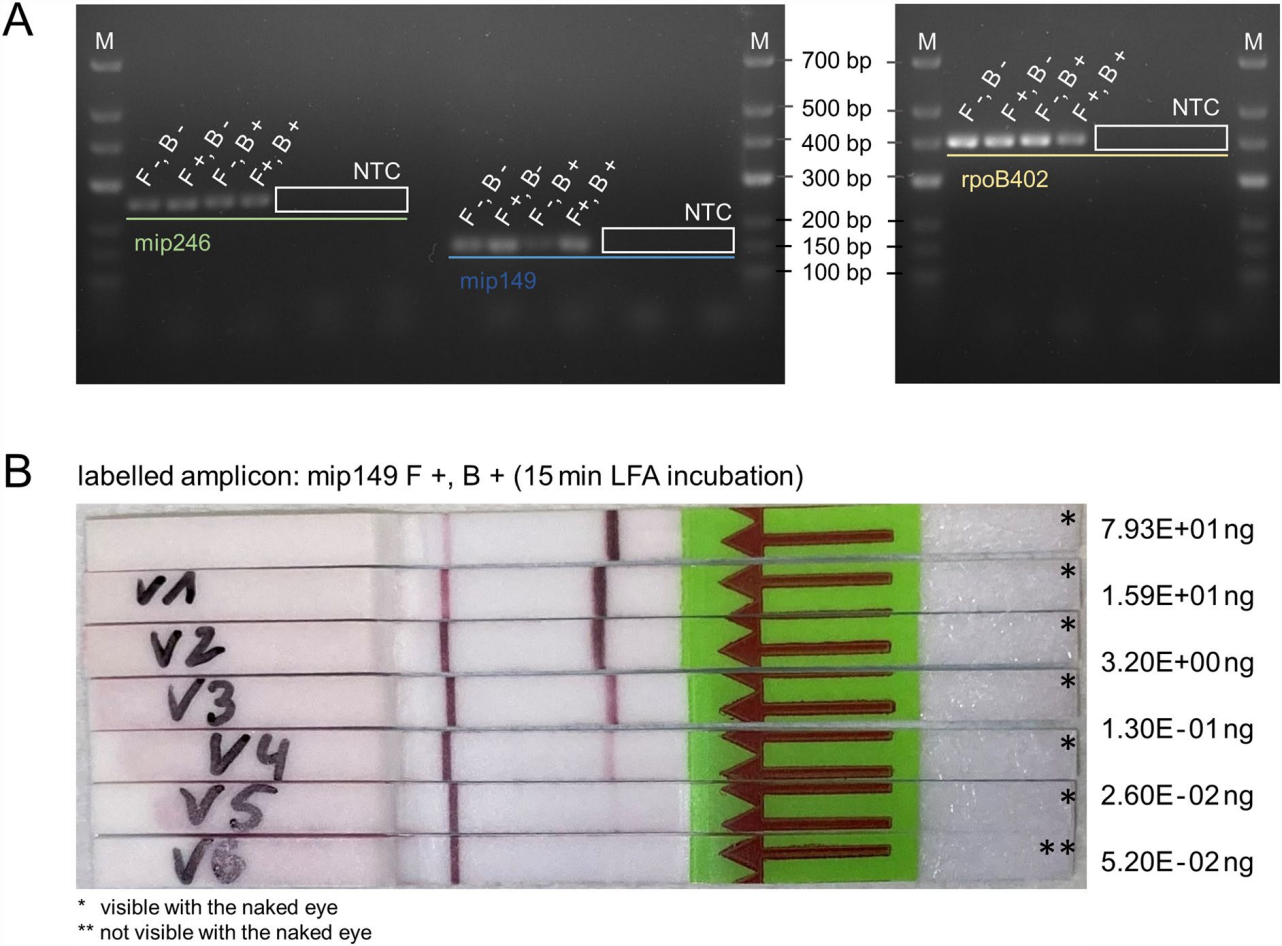
Gel electrophoresis after amplification of various gene regions from *Legionella pneumophila* and subsequent LFA detection of the mip149 amplicon. (**A**) Amplification products of different genes and sequence lengths are shown both with and without labelled primers (*F− *unlabelled forward primer, *F*+ forward primer labelled with FITC, *B− *unlabelled reverse primer, *B*+ reverse primer labelled with biotin, *NTC* no template control, *M* molecular weight marker, the numbers of the fragment names correspond to the fragment lengths to be expected). (**B**) Detection of the mip149 amplicon after labelling with FITC and biotin primers. To determine the detection limit of the test strips, the original PCR product (uppermost test strip) was diluted and analysed as a concentration series (V1 to V6) by LFA.

The aim of the study presented here was to determine whether nucleotide-based labelling of PCR products for LFA analysis represents an advantage over the commonly used primer labelling. For this purpose, an analysis was carried out using labelled primers in the first step in order to determine the influence of primer labelling on the PCR reaction and the LFA detection limit. The results are shown in Fig. 2. In the follow-up study, these findings will be compared and validated with nucleotide labelling.

Three different gene segments (mip149, mip246 and rpoB402) were amplified with four different primer combinations (double-labelled, single-labelled or unlabelled). For all samples, regardless of the primer combination, there was one band visible in the gel. All four primer combinations of a fragment (e.g. the fragment mip246) had nearly the same band position in the gel. There is a slight tendency that with increasing labelling (single to double), the respective bands are found slightly above the unlabelled products on the gel. The length of the fragments, which could be determined with the aid of the molecular weight marker (M), was approx. 250 bp for mip246, 150 bp for mip149 and 400 bp for rpoB402. None of the no template controls (NTC) were detectable in the gel. Among the variants of one fragment amplified with the means of different primer combinations, the band intensities to be observed were mainly equally distributed. Slightly weaker intensities can be seen here for the sample mip149 (F− B+) and rpoB402 (F+ B+). However, since these differences in intensity were not reproducible in consecutive experiments, we do not assume an effect of labelling. No further differences in performance were observed with these samples during the course of the study.

The analysis of the purified, undiluted PCR product (7.93E+01 ng) on the LFA (Fig. 2B) reveals a clearly recognisable test line with a weak control line. As the concentration of purified PCR-products added to the test strip decreases, the intensity of the test line also decreases while the control line increases. The lowest amount of primer-labelled PCR product visible by the naked eye is 2.6E−02 ng.

### Detection limit of LFA using nucleotide-based labelling

This study investigated whether and to what extent amplicons labelled with conjugated nucleotides are detectable on the lateral flow assay test strip. The focus thereby was on the question of which labelling method allows the lowest detectable amount of PCR product. The labelling methods applied throughout the whole study included the use of fluorescein- or biotin-conjugated nucleotides (in different concentrations) and a combination of biotin-labelled reverse primers and fluorescein nucleotides.

**Figure 3:**
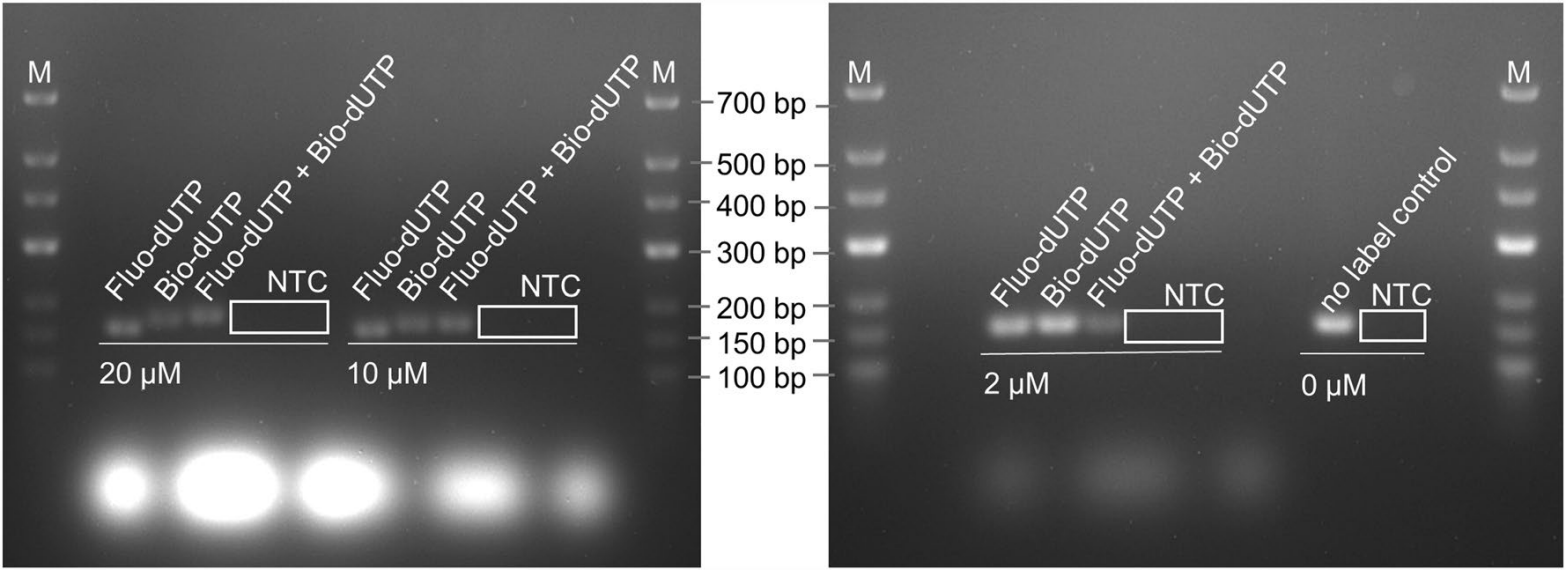
Gel electrophoresis after amplification of the gene fragment mip149 from *Legionella pneumophila* with fluorescein- or biotin-conjugated nucleotides. For labelling, different concentrations (2–20 µM) of the labelled dUTPs were used in the PCR reaction (*Fluo-dUTP* fluorescein-conjugated nucleotides, *Bio-dUTP* biotin-conjugated nucleotides, *NTC* no template control for each primer combination, *M* molecular weight marker).

In order to be able to detect or exclude a potential influence of the usage of labelled nucleotides already during PCR amplification, the amplicons were examined beforehand by means of gel electrophoresis (Fig. 3).

For this purpose, the primer combination for mip149 was used in each case using unlabelled and labelled nucleotides, the latter in different concentrations. The nucleotides, which were fluorescein- or biotin-labelled, were used both together and also separately in concentrations of 2 µM to 20 µM per conjugated dUTP in the PCR.

All samples amplified in this way showed a band at about the level of 150 bp in the gel image. The corresponding no template controls revealed no bands in the gel.

As before with primer-based labelling, the individual samples showed only very slight differences in intensity. Only the band of 2 µM fluorescein-dUTP in combination with 2 µM biotin-dUTP showed a slightly weaker signal. Here too, the handling of the PCR products certainly had a greater influence on the signal intensity than the use of the modified nucleotides, as this effect was not recognizable in the 10 µM and 20 µM samples. It is recognisable that the samples with the same nucleotide concentration do not lie on one line, but are “gradually” staggered. The PCR samples amplified with fluorescein nucleotides only, have migrated furthest in the gel. The samples with both forms of nucleotides have migrated the least far. This effect is seen most strongly in the amplicons where a nucleotide concentration of 20 µM was used and decreases in the samples with lower concentrations of labelled nucleotides.

Furthermore, bright unsharp bands can be seen at the bottom of the gel (below the 100 bp band of the molecular weight marker). These are most pronounced among the bands of samples amplified with the highest concentration of fluorescein nucleotides. The same can be observed for the corresponding NTCs. Samples amplified with biotin nucleotides only, do not show these bright spots. The intensity with which these fuzzy bands glow decreases with the concentration of fluorescein nucleotides added to the reaction mix.

**Figure 4:**
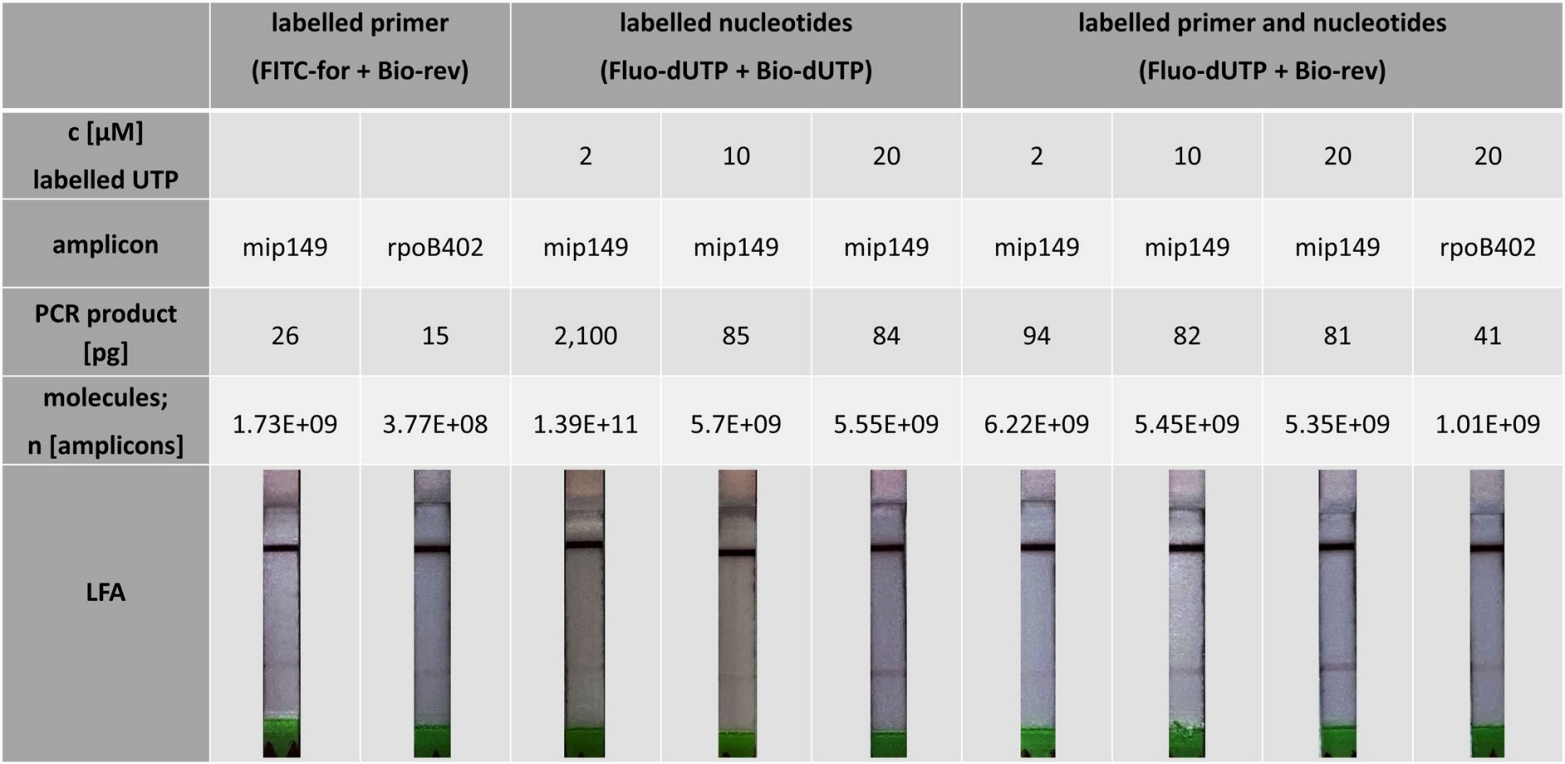
LFA-comparison of the different labelling methods. The labelling of the amplicons was performed both exclusively with primers or with nucleotides and in combination of the two methods. The rows show the concentration of the labelled nucleotides used (c [µM]) for amplification of the different fragment lengths (amplicon). The minimum amount of PCR product [pg] and the resulting amount of substance of the fragments of different lengths (number of amplicons) that was necessary to be able to recognise a signal on the LFA with the naked eye were determined. The images of the LFA strips were digitally processed in terms of contrast, exposure and sharpness in such a way as to make the weak test strip visible on the digital images (see “Methods” section).

Following PCR and purification of the amplicons, a dilution series of the differently labelled amplicons was prepared according to the LFA experiments shown in Fig. 2. Subsequent analysis on the LFA determined the lowest detectable amount of DNA based on the labelling method (Fig. 4).

Almost all labelling methods tested (except 2 µM fluorescein-dUTP in combination with 2 µM biotin-dUTP) detected amplicon amounts below 100 pg. The lowest detectable DNA (purified PCR product) amount, across all labelling methods, was 15 pg or 3.77E+08 DNA molecules of the rpoB402 fragment labelled with FITC forward primers and biotin reverse primers. Using the same labelling method, 26 pg PCR product (1.73E+09 DNA molecules) was obtained from the shorter mip149 fragment.

The highest detection limit of amplified DNA (2100 pg PCR product) to achieve a positive result on the LFA strips had to be used for the sample amplified exclusively with conjugated nucleotides at a concentration of 2 µM per nucleotide. For the amplicons labelled with higher concentrations of nucleotides (10 µM and 20 µM), 85 pg and 84 pg of DNA could be found, respectively.

In the labelling method that consisted of a combination of biotin reverse primers and different concentrations of fluorescein nucleotides, the sample with 2 µM conjugated nucleotides was also the one that had the highest detection limit with 94 pg. Nevertheless, significantly less PCR products could be used with this method than with the sample amplified with only 2 µM conjugated nucleotides (fluorescein and biotin nucleotides). The detection limit of the samples with 10 µM and 20 µM fluorescein nucleotides (in addition with biotin reverse primers) was again in the same order of magnitude as when using only labelled dUTPs. The lowest detectable amount of amplified DNA (42 pg) was again achieved with this labelling method by the longer fragment rpoB402.

#### LOD comparison of the overall process using different labelling methods

**Figure 5:**
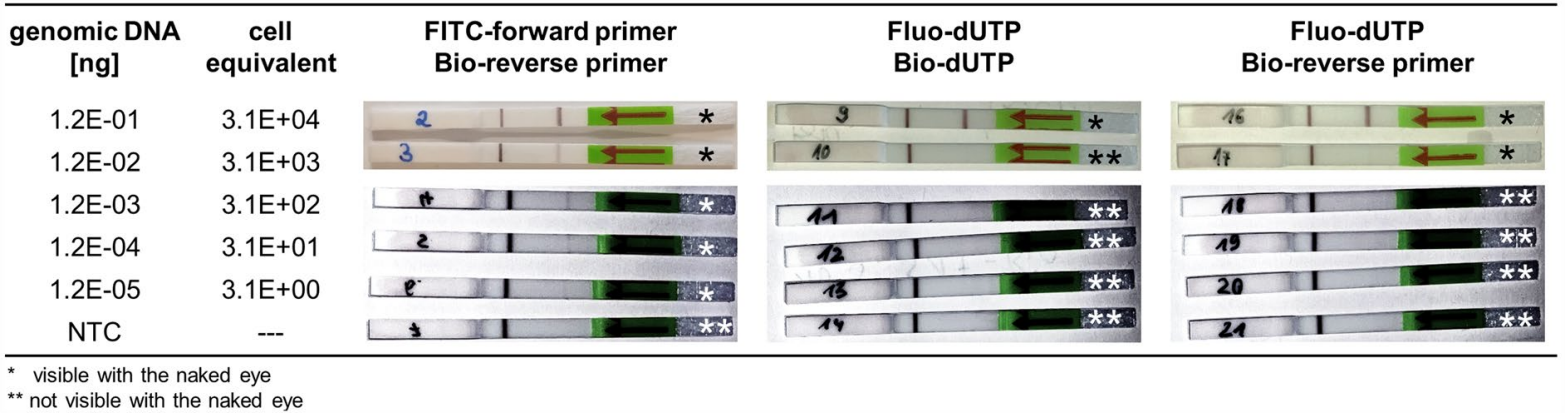
Detection limit of the LFA-PCR assay with three different labelling variations. Shown are the LFA strips for the detection of a 25 cycle PCR with different initial concentrations of genomic DNA during amplification. The samples which were amplified with FITC-forward primer/Bio-reverse primer were added to the test strips directly after PCR. All other samples of different labelling methods were purified with magnetic beads before LFA analysis. The images of the LFA strips (NTC to 1.2E−03 ng genomic DNA) were digitally processed in terms of contrast, exposure and sharpness in such a way as to make the weak test line visible on the digital images (see “Methods” section). *NTC* no template control.

To determine whether nucleotide-based labelling has advantages over the primer-based method in the case of LFA-PCR, decreasing concentration series were amplified by PCR with 25–35 cycles and analysed via LFA. The results of the 25-cycle PCR (Fig. 5) show unambiguous and clearly recognisable test bands for the concentrations 1.2E−01 ng to 1.2E−02 ng for primer labelling and exclusively 1.2E−01 ng for the other two labelling methods.

In the case of the combined labelling method (Fluo-dUTP/Bio-primer), a very weak band was still visible to the naked eye. All subsequent concentrations could not be detected on the LFA. The Fluo-dUTP/Bio-dUTP variant showed no positive results already at a concentration of 1.2E−02 ng. Both variants containing dUTP had to be purified by magnetic beads before LFA analysis. Otherwise, no test band was detectable in any of the analyses. Increasing the number of cycles to 30–35 improved the detection limit to 1.2E−04 ng of genomic DNA, but showed increased false-positive bands of the negative controls in repeated experiments (Supplementary Fig. 2).

In contrast, when labelling with primer, test bands could be detected by the naked eye after 25 cycles up to a concentration of 1.2E−05 ng genomic DNA per PCR assay and without false positive negative control. Here, too, the signal intensity could be significantly increased again at 30 cycles. In addition, the LFA detection shown here could be carried out without purification of the PCR products by direct application of the crude amplicons to the test strip.

## Discussion

The use of pre-labelled primers to label amplification products, both in PCR and in isothermal procedures, is nowadays the standard procedure for direct labelling during amplification^30–33^. Another possibility is the labelling of amplicons with nucleotides^36–38,41,44,45^. Although this is not a new method in the traditional sense and was already used in the early years of PCR^46^, it has been increasingly replaced by labelling using primers over the course of time.

Compared to primer labelling, which allows only one labelling per ssDNA the latter method can be used for multiple labelling of DNA strands. In this case, diverse marker molecules can be integrated into the DNA at the same time as well as a single marker multiple times^40,41^. The labelling efficiency mainly depends on the length of the amplicon, the nucleotide concentration used and the type of marker molecule^40^, but also on the length of linker between nucleotide and marker^47^ as well as the enzyme used for amplification^48^. While nucleotide labelling means a considerable increase in signal intensity for fluorescence-based applications, among others, the aim of this study was to clarify whether an LFA-based PCR assay (LFA-PCR) also benefits from it.

As part of this study, we looked at more than 100 PCR-LFA-based publications in our preliminary analyses. Despite the described advantages of nucleotide labelling, we could find only a single publication in which the PCR products were labelled using nucleotides^30^.

However, the assay used differs considerably from the LFA assay widely used today. Only a single label (biotin) is integrated into the amplicons. These biotin molecules are not used to immobilise the amplicons on the LFA, but to detect them using streptavidin-gold nanoparticles. Detection today is usually carried out using FITC labelling (or similar ones) and recognition using antibody-conjugated gold nanoparticles. The immobilisation of the PCR products (and isothermal products) is carried out via hybridisation to a capture probe. The sensitivity improvements achieved in the study by Kortli et al., by incorporating biotinylated dUTPs therefore do not necessarily mean a general improvement in LFA detection using conventional methods. As nucleic acid-based detection using LFA is becoming more and more important, the aim of this study was to present a comparison between the two labelling methods and to list their advantages and disadvantages.

For this reason, PCR fragments of varying lengths (150 bp to 402 bp) were labelled during amplification with primers as well as nucleotides and a combination of both. The detection limit was determined and evaluated in relation to the LFA strip itself as well as the overall assay.

As already known from other studies and shown again here (Fig. 1A), the incorporation of the pre-labelled primer does not influence the PCR amplification to any relevant extent (Fig. 1A), regardless of the length of the PCR fragment. Thus, all *Legionella*-specific dsDNA amplicons could be labelled by primer labelling with either a biotin or FITC and a combination of both.

From previous studies using LAMP and RPA as isothermal amplification methods, however, we already know that an excessive amount of labelled nucleotides can inhibit amplification^30,40,49^. This effect can also be observed here in the case of PCR (Fig. 3). The use of 2 µM Fluo-dUTP/Bio-dUTP had hardly any influence on the band intensity in the gel image and thus on the amount of amplification product. In contrast, the corresponding amounts of product decreases when using 10 µM and 20 µM [label]-dUTP. This inhibitory effect of increased concentrations of labelled nucleotides is not unknown and has already been demonstrated in early studies^30,41,50^. Prior to each study, it should be therefore determined which concentration of labelled nucleotides can be used for the specific case. No general statement can be derived here, as various factors such as the type or position of labelling, but also the type of labelled nucleotide or the enzyme used plays a crucial role^30,51,52^.

However, an increase in labelling efficiency is apparent, recognisable by the offset gel bands with 20 µM [label]-dUTP compared to 2 µM. This effect of the gel band shift was also observed in the work of Kortli et al., our own isothermal-based studies^39,41^. In general, it can be stated that [label]-dUTP concentrations in the range specified here does not represent a problem for the PCR amplification. Therefore, the detection limit of the test strip of all three variants was compared with conventional primer labelling. In addition, combinations of primer and dUTP labelling were also included in the investigation (Fig. 4).

For this purpose, the amplicons were post-processed after PCR using bead-based purification and then analysed in downward dilution series for the last band still visible to the naked eye.

In this case too, the test bands were significantly more visible with the same amount of DNA in the samples with higher [label]-dUTP concentrations during PCR. This effect occurs both in the samples labelled with Fluo-dUTP/Bio-dUTP and in the samples labelled with Fluo-dUTP/bio-primer. Thus, theoretically, an increase in signal intensity is also possible in this case by increasing the incorporation rates of marker molecules. The detection limit was 80–95 ng for almost all variants of nucleotide labelling. Only the 2 µM Fluo-dUTP/Bio-dUTP variant deviated considerably from this.

This concentration does not appear to be sufficient to statistically integrate at least one label per amplicon into the products. This may be due, among other things, to the fact that the fragment used here is a relatively short 140 bp fragment. We have already observed similar effects in earlier studies on the labelling of isothermal amplification products^40,41^. Therefore, the detection limit on the LFA here is only 2100 pg of purified PCR product and, with this amount of DNA, forces the same band intensity on the LFA strip as the much lower amounts of DNA required after using 10–20 µM nucleotides for labelling.

In comparison, however, detection limits of 15–25 pg could be achieved with the primer labelling for both the short (150 bp) and the long (402 bp) PCR fragments. Thus, it seems that the primer labelling achieves a slightly better detection limit than the nucleotide-based one.

This observation contradicts our previous expectations and also the previously mentioned improvements in sensitivity observed by Kortli et al., through the use of nucleotide labelling.

In other studies presented, the incorporation of fluorescent molecules into isothermal products through the use of labelled nucleotides led to improved incorporation rate of the labels depending on the fragment length and nucleotide concentration as well as the amplification method used, thereby increasing sensitivity^40,42,41^. In isothermal studies, we were able to show that an incorporation number of 2–3 nucleotides is achieved when using 20 µM labelled nucleotides in recombinase polymerase amplification (RPA)^41^. In the case of the study shown here and based on our expertise in PCR labelling, we also assume an incorporation rate of 2–3 labels for fragment lengths between 150 and 500 bp, although this was not explicitly determined here. In the case of PCR-LFA detection, however, further effects appear to reduce the advantages of nucleotide labelling.

Whether primer labelling finally has advantages over nucleotide labelling within a real assay was investigated using a concentration series of genomic *Legionella*-DNA during PCR and simultaneous labelling.

It was shown that by using primer, a test band up to a concentration of 1.2E−05 ng (corresponding to 3.1E+00 cell equivalents) was just visible to the naked eye after a 25 cycle PCR. The intensity of the bands increased considerably at 35 cycles. Here, the amplicons could be analysed directly after PCR without further post-treatment with the test strip. This means that no purification steps were necessary. This was not possible when using the nucleotide-labelled variants with 20 µM [label]-dUTP each (Supplementary Fig. 1). The samples had to be cleaned of unused and excessive Fluo-dUTP or Bio-dUTP after amplification using bead-based purification. Otherwise, the remaining excess of Bio-dUTP would occupy the binding sites on the test strip and no target DNA could be bound. Furthermore, the detection antibodies were intercepted by the free Fluo-dUTP and in turn prevented detection of the amplicons. Taken together, the unbound, labelled nucleotides compete with the labelled PCR products for the free binding sites on the streptavidin and the detection antibodies. This was noticeable by the fact that a positive test band appeared in the first minute of LFA detection, which became negative again after nearly seven minutes (Supplementary Fig. 1). This effect did not occur if the nucleotide-labelled PCR products were purified before analysis.

As a result, the nucleotide-labelled amplicons could only be detected via lateral flow detection with considerable additional effort.

When comparing the purified primer labels with the purified nucleotide labels, there was also a considerable difference in the limit of detection (LOD). While the former, as already described, was 3.1E+00 cell equivalents, at least 3.1E+03 to 3.1E+04 cell equivalents (1.2E−02 to 1.2E−01 ng genomic DNA) were required for PCR for both the Fluo-dUTP/Bio-primer and Fluo-dUTP/Bio-dUTP variants. Furthermore, no increase in sensitivity by increasing the number of cycles in the PCR was possible in the same way as it was performed with primer labelling, achieved an improvement. Here, the aspect that valuable sample material is lost due to the mandatory post-processing of the PCR products certainly also comes into play.

Therefore, it can be summarised at this point that labelling of amplicons for detection by LFA can in principle be carried out by dNTPs instead of primers, or a combination of both, but it is less sensitive in the case of PCR amplification. The advantage here seems to be clearly on the side of the primer labelling.

It should be noted that this statement seems true for a simple singleplex assay. There are various studies that show that multiplex analyses using PCR-LFA are also technically possible^53–55^. Apart from the fact that the test strip design has to be extended for this, e.g. for binding via digoxigenin (dig) in addition to biotin^54,55^, false positives could be detected if one or more of the targets are not present and have therefore not been amplified. In this case, two basic scenarios are conceivable: (i) the unused primers bind to the surface of the test strip, but since there is no binding site for the detection antibody (by the FITC-primer), they do not generate a signal. We have also been able to observe this result here on our NTCs. (ii) The absence of target DNA or poorly matched primer design could favour the amplification of primer dimers or incorrect amplification products due to unspecific primer bindings, for example. The resulting products in turn would carry both markers and would therefore be detected as false positives^56^. However, this question must certainly be tested in each individual case with the specific primer combinations and earlier research have already shown that multiplex analyses are feasible with reasonably designed primers^32,33^.

This is another advantage of primer-based labelling for this detection method. Different molecules such as biotin and digoxigenin can be specifically integrated into different sequences, whereas nucleotides are incorporated non-specifically and sequence-independently. A multiplex assay with nucleotides would therefore be rather difficult to implement.

However, in some cases of isothermal amplification, nucleotide labelling also seems to work perfectly. Agarwal et al.^44^ and Zhang et al.^38^ were able to demonstrate successful and sensitive detection (> 2 viral copies/LAMP assay) of LAMP products using a combination of primer and nucleotide labelling. In the case of our PCR analyses, detection of *Legionella*-DNA was also possible with this labelling combination. Unfortunately, with 3.1E+03 cell equivalents, this had a lower detection limit than pure primer labelling, but still a higher one than with pure nucleotide labelling. In the case of the LAMP however, the amount of produced DNA during amplification can be many times greater than with PCR. This could possibly cause the following effects. Firstly, the increased amplification of DNA product consumes more of the freely available labelling dNTPs. Thus, these can interfere less on the LFA strip. Secondly, the higher amount of DNA produced can compete more effectively for the binding sites on the test strip. Therefore, in the case of LAMP, it was certainly possible to detect the amplicon sensitively even without downstream purification.

Thus, depending on the amplification method (PCR or isothermal), detection method (e.g. gel electrophoresis, LFA or microarray) and concentration of the labelling dNTPs, nucleotide labelling certainly has advantages with respect to the labelling efficiency of the DNA strands. However, this advantage cannot be exploited in all cases such as the LFA-PCR detection presented here. Therefore, in the case of PCR, the labelling of the amplicons by means of primers is still recommended according to this study.

## Methods

### PCR reaction and amplicon labelling

Unless stated otherwise, PCR was performed according to the manufacturer’s instructions using the Phusion High-Fidelity PCR kit (Thermo Fisher Scientific Inc; F553L). For this purpose, the final 25 µL PCR mix contained 1× Phusion HF buffer, unlabelled dNTPs (200 µM each), Phusion DNA polymerase (0.5U), unlabelled forward and reverse primer (0.5 µM each), template (0.04E+00 to 4.80E−07; the corresponding concentrations are given in the results section). For the labelling of amplicons by primer, all unlabelled forward and/or reverse primer were replaced by labelled primer. For labelling with biotin-11-dUTPs (Jena Bioscience GmbH; NU-803-BIOX-S) or fluorescein-12-dUTPs (Jena Bioscience GmbH; NU-803-FAMX-S), the corresponding amounts (2 µM to 20 µM per 25 µL PCR) were added to the existing PCR-mixture.

The amplification with and without labelling took place as follows: (i) initial denaturation (98 °C for 30 s); (ii) denaturation (98 °C for 10 s); (iii) annealing (60 °C for 30 s); (iv) elongation (72 °C for 30 s); repeat steps ii-iv for 24–34 cycles; (v) final elongation (72 °C for 10 min); (vi) cooling (4 °C for 1 h).

The organisms and primers listed in Table 1 were used for the PCR analyses. The purified genomic DNA was provided by the *Konsiliarlaboratorium für Legionellen*, University Hospital Carl Gustav Carus Dresden (UKD). The primers were obtained from Eurofins Genomics Germany GmbH.

**Table 1:** List of organism and primer.

Organism	NCBI Ref. Seq.	NCBI gene ID	Name	Primer	Sequence	[bp]
*Legionella pneumophilia* str. Corby	NC_009494.2	*mip* (66489975)	mip149	P_mip_450F	ATATACTGGTCGTCTGATTGAT	149
				P_mip_599R	GGAACATAAATTTCCCAAGTTG	
		*mip* (66489975)	mip246	P_mip_77F	CATTAGCTACAGACAAGGATAA	246
				P_mip_323R	CCTTTTACTTTATTTTCATCCGC	
		*rpoB* (66489521*)*	rpoB402	P_rpoB_681F	CTACGAAGCAGAAGATATATTAA	402
				P_rpoB_1083R	GTAAGAACCATGATCCAAATCA	

Illustration of the organism and primer used in this study. All primer were synthesized and provided by Eurofins Genomics Germany GmbH. For labelling, the forward primers were pre-labelled with FITC and reverse primers with biotin.

The genomic DNA of *L. pneumophilia* was prepared and provided by the *Konsiliarlaboratorium für Legionellen*, University Hospital Carl Gustav Carus Dresden (UKD).

### Analysis by gel electrophoresis

Following the PCR, samples were tested for amplification success by gel electrophoresis using a 2% agarose gel (Sigma-Aldrich; A6877) in TAE buffer (AppliChem GmbH; A1691) at 4 °C. With a volume of 100 ml, the gel contained 4 µL of the dye peqGreen (VWR International GmbH; 37–500). For the loading of the agarose gel, 5 µL of the unpurified PCR product was mixed with 1 µL of 6× DNA Loading Dye (Thermo Fisher Scientific Inc.; R0611). The molecular weight marker used was composed of 1 µL Gene Ruler Low Range DNA Ladder (Thermo Fisher Scientific Inc.; SM1191), 1 µL 6 × TriTrack DNA Loading Dye (Thermo Fisher Scientific Inc.; R1161) and 4 µL nuclease-free water. Electrophoresis ran at a constant voltage of 100 V for 90–120 min (depending on the volume of the gel). The gel electrophoresis results were documented using the Quantum CX5 gel documentation system from Vilber Lourmat Deutschland GmbH (Eberhardzell, Germany) and the related BioVision software.

### Lateral flow assay

PCR products labelled with nucleotides were purified with the Mag-Bind® Total Pure NGS Kit (Omega Bio-Tek; M1378-01) according to the manufacturer's instructions prior to LFA analysis to determine the test strip detection limit. Afterwards, elution with 20 µL ddH_2_O was performed. The remaining LFA experiments (i.e. primer labelling) were performed with unpurified amplicons immediately after PCR. For this, 90 µL of the corresponding HybriDetect assay buffer was added to one well of a 96-well plate to perform the HybriDetect Universal Lateral Flow Assay (Milenia Biotec GmbH; MGHD 1). 10 µL of the sample to be analysed was placed on the sample pad of the test strip. Afterwards, with the sample pad facing downwards, the test strip was placed in the prepared well containing the HybriDetect Assay Buffer. The test was removed from the well after 15 min incubation and evaluated. The result of the test strip was considered positive if it showed two lines (control line and test line). If only the control line was visible, the result was negative. If the control line was missing, the test result was invalid. The documentation of the result was done with a smartphone camera (Apple Iphone SE; second generation).

### Image editing

The images of the lateral flow assay test strips were retrospectively edited for presentation in Figs. 4 and 5, only. For this purpose, the image editing function of the Microsoft Photos app was used. The default values were changed to the following settings: (i) contrast 100%, (ii) exposure − 50%, (iii) sharpness 100%. The remaining parameters were left unchanged if not mentioned in further detail.

### Concentration measurements

Concentration measurements were conducted using Nanodrop 2000 (Thermo Fisher Scientific) with 1.5 µL purified PCR products.

The concentration of genomic DNA was determined in the same way by measurement at 260 nm on the Nandrop 2000.

An average genome size of 3.6 Mbp (*Legionella pneumophila* str. Corby; NCBI Reference Sequence: NC_009494.2) with a molar mass per base pair of 650 (g/mol)/bp^57^ was assumed for the conversion of the concentration of genomic DNA used into cell equivalents.

### Supplementary Information

Supplementary Figures.

## Data Availability

The data that support the findings of this study are available from the corresponding author [C.W.] upon reasonable request.
