# Supplementary material for: Comparison of pre-labelled primers and nucleotides as DNA labelling method for lateral flow detection of Legionella pneumophila amplicons
Source: Sci Rep. 2024 Feb 29;14:5018. doi: 10.1038/s41598-024-55703-4 (PMC10904838; doi:10.1038/s41598-024-55703-4)
Supplement: Supplementary file 1 — Supplementary Figures. [file 41598_2024_55703_MOESM1_ESM.pdf]

Comparison of pre-labelled primers and nucleotides as DNA labelling method for lateral flow detection of *Legionella pneumophila* amplicons.

Christian Warmt[1,*], Jette Nagaba[1], Jörg Henkel[1]

[1]Fraunhofer Institute for Cell Therapy and Immunology - Bioanalytics and Bioprocesses (IZI-BB), 14476 Potsdam, Germany

* christian.warmt@izi-bb.fraunhofer.de

Supplementary Information

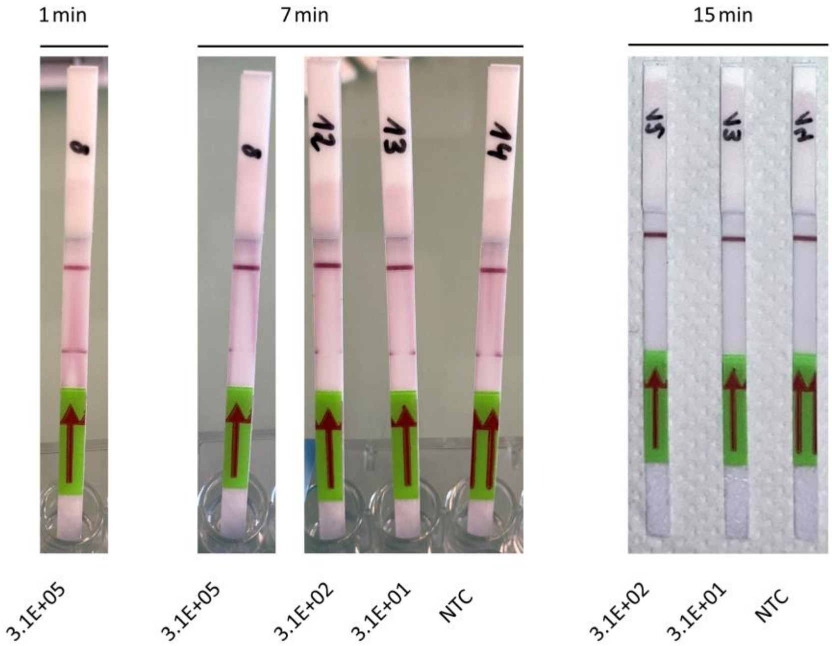

Supplementary Figure 1: Impossible detection of unpurified PCR products after labelling with nucleotides. The PCR products were labelled with biotin-dUTP and FITC-dUTP. During the first minute of incubation of the test strip, a positive test line appears as usual, which disappears in the course of the incubation and is no longer recognizable after 15 min. (right figure mirrored for uniform representation)

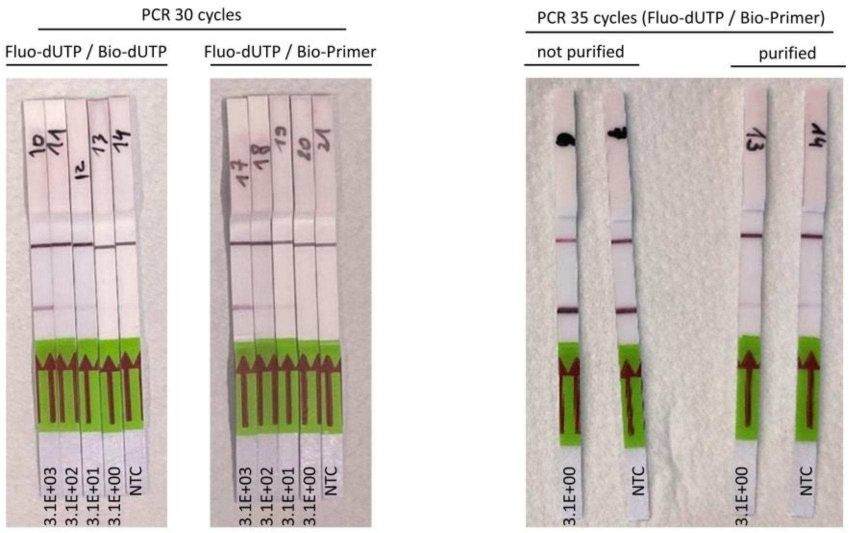

Supplementary Figure 2: False-positive results when increasing the number of PCR cycles. Although increasing the number of PCR cycles to 30-35 cycles generally increases the sensitivity of the entire assay, it also increases the risk of false-positive results.
